# Corticotropin Releasing Factor (CRF) Coexpression in GABAergic, Glutamatergic, and GABA/Glutamatergic Subpopulations in the Central Extended Amygdala and Ventral Pallidum of Young Male Primates

**DOI:** 10.1523/JNEUROSCI.1453-22.2022

**Published:** 2022-11-30

**Authors:** Julie L. Fudge, Emily A. Kelly, Troy A. Hackett

**Affiliations:** ^1^Del Monte Institute for Neuroscience, University of Rochester, Rochester, NY 14642; ^2^Vanderbilt University Medical Center, Nashville, TN 37232

**Keywords:** basal forebrain, bed nucleus of the stria terminals, central nucleus, globus pallidus, nonhuman primate, substantia innominata

## Abstract

The central extended amygdala (CEA) and ventral pallidum (VP) are involved in diverse motivated behaviors based on rodent models. These structures are conserved, but expanded, in higher primates, including human. Corticotropin releasing factor (CRF), a canonical “stress molecule” associated with the CEA and VP circuitry across species, is dynamically regulated by stress and drugs of abuse and misuse. CRF's effects on circuits critically depend on its colocation with primary “fast” transmitters, making this crucial for understanding circuit effects. We surveyed the distribution and colocalization of CRF-, VGluT2- (vesicular glutamate transporter 2), and VGAT- (vesicular GABA transporter) mRNA in specific subregions of the CEA and VP in young male monkeys. Although CRF-containing neurons were clustered in the lateral central bed nucleus (BSTLcn), the majority were broadly dispersed throughout other CEA subregions, and the VP. CRF/VGAT-only neurons were highest in the BSTLcn, lateral central amygdala nucleus (CeLcn), and medial central amygdala nucleus (CeM) (74%, 73%, and 85%, respectively). In contrast, lower percentages of CRF/VGAT only neurons populated the sublenticular extended amygdala (SLEAc), ventrolateral bed nucleus (BSTLP), and VP (53%, 54%, 17%, respectively), which had higher complements of CRF/VGAT/VGluT2-labeled neurons (33%, 29%, 67%, respectively). Thus, the majority of CRF-neurons at the “poles” (BSTLcn and CeLcn/CeM) of the CEA are inhibitory, while the “extended” BSTLP and SLEAc subregions, and neighboring VP, have a more complex profile with admixtures of “multiplexed” excitatory CRF neurons. CRF's colocalization with its various fast transmitters is likely circuit-specific, and relevant for understanding CRF actions on specific target sites.

**SIGNIFICANCE STATEMENT** The central extended amygdala (CEA) and ventral pallidum (VP) regulate multiple motivated behaviors through differential downstream projections. The stress neuropeptide corticotropin releasing factor (CRF) is enriched in the CEA, and is thought to “set the gain” through modulatory effects on coexpressed primary transmitters. Using protein and transcript assays in monkey, we found that CRF neurons are broadly and diffusely distributed in CEA and VP. CRF mRNA^+^ neurons colocalize with VGAT (GABA) and VGluT2 (glutamate) mRNAs in different proportions depending on subregion. CRF mRNA was also coexpressed in a subpopulation of VGAT/VGluT2 mRNA (“multiplexed”) cells, which were most prominent in the VP and “pallidal”-like parts of the CEA. Heterogeneous CRF and fast transmitter coexpression across CEA/VP subregions implies circuit-specific effects.

## Introduction

The central extended amygdala (CEA) and ventral pallidum (VP) mediate anxiety and defensive responses, as well as appetitive behaviors, all of which can be altered in stressful situations (Root et al., 2015; Rinker et al., 2017; Giardino et al., 2018; Soden et al., 2022). As such, these structures are considered important in regulating homeostatic stress responses (Fox et al., 2015). In human and nonhuman primates, the CEA includes the lateral bed nucleus of the stria terminalis (BSTL), the central nucleus of the amygdala (CeN), and the cell columns that stretch between them (known as the central sublenticular extended amygdala [SLEAc]) (Alheid and Heimer, 1988; Heimer et al., 1999). The overlying VP, a closely related transition region, stretches caudally following the path of the SLEAc.

The CEA contains some of the highest neuropeptide levels in the brain (Price et al., 1987; Martin et al., 1991; Walter et al., 1991; Fox et al., 2015). One of these neuropeptides, corticotrophin releasing factor (CRF), is a neuroregulator of the stress response and contributes to the regulation of specific neural circuits (Kelly and Fudge, 2018). CRF neurons in the CEA have been a longstanding focus for understanding how “stressful” emotional experiences can directly influence downstream effector sites to shape defensive and motivated behaviors (Erb et al., 2001; Lee et al., 2008; Zorrilla et al., 2014; Pleil et al., 2015).

Like most peptides, CRF is always expressed in concert with primary “fast” transmitters (e.g., glutamate or GABA) (Orozco-Cabal et al., 2006; Gallagher et al., 2008; Joels and Baram, 2009), and acts with them to enhance their actions at the synapse. CRF coexpression shapes postsynaptic responses, depending on the primary transmitter involved (Orozco-Cabal et al., 2006; Partridge et al., 2016). Therefore, the distribution of CRF with its primary transmitters in specific CEA and VP territories has major implications for how it enhances or dampens signals in specific outputs to downstream effector sites. While GABAergic neurons were long assumed to be the primary cell type in the CEA and VP (Parent et al., 1995; McDonald, 2003), the discovery of the glutamate-specific vesicular transporters has permitted a more nuanced picture (Fremeau et al., 2001, 2004). Based on ISH, glutamatergic neurons (VGluT2-positive) are found in subregions of the CEA and pallidum in rodents (Hur and Zaborszky, 2005). Moreover, specific neuropeptides have been colocalized in GABAergic and/or glutamatergic cell populations across the region (Poulin et al., 2008, 2009; Kudo et al., 2012; Partridge et al., 2016; Soden et al., 2022).

Although some data on CRF expression in specific GABAergic and glutamatergic neuronal populations and circuits are emerging in mice and rats, data in higher primates are lacking. In addition, while there is some conserved organization of the CRF systems between the primate, rat, and mouse models, there are also important differences (Kelly and Fudge, 2018; Kovner et al., 2019, 2020). Structurally, the primate CEA has fewer discrete subnuclear regions compared with rodents (De Olmos, 1990; Martin et al., 1991; Heimer et al., 1999), which is reminiscent of species differences in the hypothalamus (Koutcherov et al., 2000). Accordingly, CRF neuron distribution in the CEA appears more diffuse than in rodent (Bassett and Foote, 1992; Kelly and Fudge, 2018; Kovner et al., 2020).

The current paper examines the neuroanatomic distribution of the CRF immunoreactive neurons in the CEA and VP, compares CRF protein and transcript distribution, and then quantifies the extent of CRF mRNA colocalization with its primary transmitters along this trajectory. Understanding CRF's role with its primary transmitter(s) in primate extended amygdala is a critical first step for understanding CRF's modulatory role in specific stress circuitry, which is relevant for human neuropsychiatric disorders (Sanders and Nemeroff, 2016).

## Materials and Methods

### Tissue preparation for immunocytochemistry (ICC) and ISH

We used 3 young male *Macaca fascicularis* (MFJ26, MFJ17, and MFJ38) to examine CRF-IR-labeled cells through the CEA subdivisions (ages 3.0, 3.0, and 3.3 years, respectively). Following ICC confirmation of similar CRF protein expression in the ROIs in all animals, tissue from 1 of the animals (MFJ38) was selected for ISH studies. All animal use was in accordance with the National Institutes of Health's *Guide for the care and use of laboratory animals*, and with the University of Rochester's Institutional Animal Care and Use Committee. All animals were pair- or group-housed and maintained on a 12 h light-dark cycle. Two of the animals (MFJ26 and MFJ17) were previously used in tract tracing experiments, and MFJ38 was a normal control. Tissue from MFJ38 was also used for ISH. Animals were deeply anesthetized and killed by perfusion through the heart with 0.9% saline containing 0.5 ml of heparin sulfate (200 ml/min for 10 min), followed by cold 4% PFA in a 0.1 m PB/30% sucrose solution (100 ml/min for 1 h). The brain was extracted from the skull, placed in a fixative overnight, and then put through increasing gradients of sucrose (10%, 20%, 30%). All brains were cut on a freezing microtome (40 μm), and all sections were stored in cryoprotectant solution (30% ethylene glycol and 30% sucrose in 0.1 m PB) at −20°C (Rosene et al., 1986).

### ICC

#### Defining the CEA and VP subregions using CRF, substance P (SP), somatostatin (SST), vasoactive intestinal peptide (VIP), and calbindinD-28k (CaBP) immunoreactivity (IR), and acetylcholinesterase (AChE) staining

To identify the location of the specific CEA and VP subdivisions in monkey, we used several markers that have been previously described in monkey and human (Beach and McGeer, 1984; Haber and Watson, 1985; Mai et al., 1986; Haber et al., 1990a; Cote et al., 1991; Martin et al., 1991; Walter et al., 1991; Heimer et al., 1999) (see Results). In primate CEA (including human), low AChE levels and relatively high neuropeptide staining define the oval-shaped lateral core of the bed nucleus of the stria terminalis (BSTLcn) and the central nucleus (CeLcn). The BSTLJ and amygdalostriatal areas have higher levels of the “striatal” marker AChE. The BSTLP and CeM, and their contiguity with the SLEAc, have moderate levels of AChE. They also have patches of SST- and VIP-IR that avoid the CaBP-positive globus pallidus, and can therefore serve as distinguishing markers for the SLEAc (Fudge and Haber, 2001; Kovner et al., 2019). SP-IR, usually considered a “marker” of the VP in rodents (Haber and Nauta, 1983), is not used as an exclusive marker of the primate VP and internal segment of the globus pallidus (GPi), since it is also enriched in the SLEAc (CaBP-negative region beneath the globus pallidus) (Haber and Watson, 1985; Haber et al., 1990b), and forms “wooly fibers” in each region.

#### Characterization of antibodies for ICC

We performed dilution curves of three CRF-antibodies generated to assess single-label CRF-IR, two made commercially (Peninsula, made in rabbit, T4037; Peninsula, made in guinea pig, T5007) and one raised against a 41 amino acid peptide isolated from bovine hypothalamus and pharmacologically and physiologically characterized (Olschowka et al., 1982) (Table 1). The commercial antibodies were raised against the common human/rat (h/r)CRF peptide (SEEPPISLDLTFHLLREVLEMARAEQLAQQAHSNRKLMEII-NH_2_). Dot-blot analysis in previous work indicates that these anti-CRF antibodies recognize h/rCRF (code C-3042) but do not label rat urocortin I (code U-6631), mouse urocortin II (code U-9507), or human urocortin III (code U-1008) (Tagliaferro and Morales, 2008). We also trialed the antibody created from a synthetic CRF 41 AA acid sequence, raised in rabbit (gift of John Olschowka, University of Rochester). IR to all antibodies was similar compared with earlier studies in primates (Paull et al., 1984; Foote and Cha, 1988; Lewis et al., 1989; Bassett and Foote, 1992; Kovner et al., 2019), and we selected the commercial anti-CRF antibody (Peninsula, made in rabbit, T4037), because of ready availability. We noted many labeled cells in the paraventricular and lateral hypothalamus and thalamus (not shown), consistent with previous reports in monkey (Bassett and Foote, 1992). The pattern of calbindin-D28K (CaBP) expression was identical to published reports for our ROIs, as well as in other brain areas, such as the striatum and substantia nigra (Cote et al., 1991; Meredith et al., 1996). Finally, the pattern of SP, SST, and VIP IR in the basal forebrain matched previously published results in human and monkey (Mai et al., 1987; Martin et al., 1991; Heimer et al., 1999; Fudge and Haber, 2001). These antisera were used to stain near-adjacent sections with the purpose of colocalizing CRF-IR and CRF mRNA-positive neurons in specific CEA subdivisions.

**Table 1.** Primary and secondary antibody information

Primary antibody	Immunogen	Source (catalog #)	Working dilution	Secondary antibody
Mouse monoclonal anti-CaBP	Purified bovine kidney CaBP	Sigma (C-9848)	1:10,000	Goat anti-mouse IgG, 1:200, Vector Laboratories
Rabbit polyclonal anti-SST	Synthetic human SST (amino acid sequence: Ala-Gly-Cys-Lys-Asn-Phe-Phe-Trp-Lys-Thr-Phe-Ser-Cys)	Immunostar #20067	1:1000	Goat anti-rabbit IgG, 1:200, Vector Laboratories
Rabbit polyclonal anti-VIP	Porcine VIP coupled to bovine thyroglobulin (BTg) with carbodiimide (CDI) linker	Immunostar #20077	1:5000	Goat anti-rabbit IgG, 1:200, Vector Laboratories
Rabbit polyclonal SP	Synthetic SP coupled to KLH with carbodiimide	Immunostar #20064	1:5000	Goat anti-rabbit IgG, 1:200, Vector Laboratories
Rabbit polyclonal CRF	Synthetic ovine CRF coupled to bovine thyroglobulin (1:10), with ethyl-3- (3-dimethyl-aminopropyl) carbodiimide	Gift, Dr John Olschowka	1:3000	Goat anti-rabbit IgG, 1:200, Vector Laboratories
Rabbit polyclonal CRF	Synthetic peptide H-Ser-Glu-Glu-Pro-Pro-Ile-Ser-Leu-Asp-Leu-Thr-Phe- His-Leu-Leu-Arg-Glu-Val-Leu-Glu-Met-Ala-Arg-Ala-Glu-Gln-Leu-Ala-Gln- Gln-Ala-His-Ser-Asn-Arg-Lys-Leu-Met-Glu-Ile-Ile-NH2 coupled to carrier protein	BMA Biomedicals (formerly Peninsula Labs) T-4037	1:6000	Goat anti-rabbit IgG, 1:200, Vector Laboratories
Guinea pig polyclonal CRF	Synthetic peptide H-Ser-Glu-Glu-Pro-Pro-Ile-Ser-Leu-Asp-Leu-Thr-Phe- His-Leu-Leu-Arg-Glu-Val-Leu-Glu-Met-Ala-Arg-Ala-Glu-Gln-Leu-Ala-Gln- Gln-Ala-His-Ser-Asn-Arg-Lys-Leu-Met-Glu-Ile-Ile-NH2 coupled to carrier protein	BMA Biomedicals (formerly Peninsula Labs) T-5007	1:4000	Donkey anti-guinea pig, 1:200, Vector Laboratories

1:24 sections through the CEA that were adjacent or near-adjacent to CRF-IR and CRF mRNA treated tissue were chosen for single-label ICC. For visualization of neuropeptides and CaBP proteins, adjacent sections were rinsed in 0.1 m PB-TX, preincubated in 10% NGS-PB-TX as described above, and then incubated for 96 h at 4°C in CaBP (Sigma, 1: 10,000, mouse), SP (Immunostar, 1:1000, rabbit), SST (Immunostar, 1:1000, rabbit), VIP (Immunostar, 1:1000, rabbit), and CRF (Peninsula Laboratories, 1:6000, rabbit). Sections were then rinsed, blocked with 10% NGS-TX, and incubated with the appropriate secondary biotinylated mouse or rabbit antibody. Following rinsing, the molecules were visualized using avidin-biotin reaction. AChE staining was performed in adjacent compartments using the Geneser technique (Geneser-Jensen and Blackstad, 1971).

### ISH and FISH

#### RNAScope mRNA probe selection and processing

RNAScope is a branched (“tree”) assay that allows detection of mRNA transcripts at high cellular resolution (F. Wang et al., 2012; Grabinski et al., 2015) (ACD Bio). Target probes were designed for both 2-plex colorimetric assays and 4-plex fluorescent assays. All probes were designed for macaque (Mmu) as follows: Mmu-corticotropin releasing hormone (CRH), 838961; Mmu-GAD1, glutamate decarboxylase 1 (GAD1); Mmu SLC32A1 (solute carrier family 32, GABA vesicular transporter, VGAT); Mmu SLC17A6, solute carrier family 17 (sodium-dependent inorganic phosphate cotransporter, member 6 transcript variant 2 (VGluT2); and Mmu SLC17A8 (solute carrier family 17, member 8, VGluT3) mRNA. 1:24 hemi-sections were selected and blocked to include the CEA and VP along the rostrocaudal forebrain.

We used RNAScope technology (ACD Bio) in a series of experiments using colorimetric and fluorescent applications to confirm results using several methods. We examined transcripts for both GAD1, the synthetic enzyme for GABA, and the GABA vesicular transporter (VGAT) to assess distribution of GABAergic cells. Probes for the glutamate transporters VGluT2 and VGlut3 mRNAs were also used in preliminary experiments to assess for distribution of glutamatergic cells. VGluT1 mRNA is not expressed in the CEA structures (Allen Brain Bank, nonhuman primate database). Positive and negative control regions for all probes were preliminarily assessed to ensure validity: VGluT2 (thalamus), VGluT3 (brainstem), CRF (paraventricular hypothalamus), GAD1 and VGAT (cortex, striatum).

Dual-probe colorimetric ISH and multi-FISH were conducted in floating sections using RNAscope detection kits and reagents following the manufacturer's protocols. All solutions were made with MilliQ-filtered water treated with a Biopak filter to minimize RNase and pyrogenic activity. Sections were removed from cryostorage, rinsed in 0.1 m PB, then incubated 10 min in H_2_O_2_. Sections were incubated in a citric acid-based target retrieval solution (RNAscope) for 7 min at 99°C, rinsed in MilliQ H_2_0, then permeabilized in Protease Plus (RNAscope) for 30 min at 40°C. Sections were incubated for 2 h with premixed riboprobes, rinsed in Wash Buffer (RNAscope), then reacted with the dual-probe colorimetric or multiplex fluorescence detection kit (V2) provided by RNAscope. For dual-probe colorimetric ISH, signal detection used RNAscope duplex kits (2.5 HD) containing red and green dyes for simultaneous detection of two probes. For FISH, signal detection used Akoya fluorescent dyes (Akoya Biosciences) (1:1000) using tyrosine signal amplification in up to four color channels (520, 570, 650, 780 nm). After amplification, sections were rinsed, counterstained with DAPI, mounted on glass slides, then coverslipped. Tiled images were captured using a 20× objective on a Nikon 90i epifluorescence microscope controlled by Nikon Elements software. Using the large-image scan function and automated focus, color channels were captured sequentially for each tile (filters: DAPI, 520, 570, 650, 780 nm), then stitched into a final 5-color image stack for analysis.

### Analyses

#### ICC labeling

1:24 sections through the CEA and ventral VP were charted under bright-field microscopy with a 10× objective, assisted with Neurolucida (Microbrightfield Biosciences) mapping software with automatic cell counting. Adjacent sections stained for AChE or immunoreacted for CaBP, SST, and SP proteins were also mapped to delineate nuclear boundaries of the striatum, BSTLcn, BSTLJ, BSTLP, pallidum, SLEAc, and CeM and CeLcn. Contours of these boundaries were overlaid onto CRF-IR neuronal maps in Neurolucida, carefully aligning fiducial markers, such as fiber tracts (anterior commissure, internal capsule, optic chiasm, fornix) and blood vessels. Maps were exported as SVG files, then imported, placed in transparent layers, and finalized for publication in Adobe Illustrator 2022 (www.adobe.com).

#### VGluT2/GAD1 mRNA and VGlut3 mRNA

In a series of experiments, we first assessed 1- and 2-plex colorimetric processing to determine the general expression pattern of glutamatergic and GABAergic markers in the CEA/VP, and potential colocalization. Qualitative assessments were done on tiled sections, and visualized using brightfield microscopy. VGluT2 (red) and GAD1 (blue) mRNAs were largely found in separate populations of neurons in the CEA, with VGluT2-positive neurons tending to be larger in size, and more sparsely distributed, than GAD1-positive cells. However, colocalization of VGluT2/GAD1 mRNA (purple) was noted in subpopulations of cells of the VP and GPi (see Results; Fig. 6), and in the surrounding SLEAc. In contrast, 1-plex studies showed that VGluT3 mRNA was only seen in very sparse numbers of cells in the CEA and VP, and was largely expressed in the large cholinergic cell groups of the basal forebrain (e.g., nucleus basalis of Meynert; see Fig. 7) (Gras et al., 2002).

#### FISH for CRF-, VGAT-, and VGluT2-mRNA

Based on 1- and 2-plex mRNA labeling experiments, we focused on 4-plex fluorescent analyses on VGAT, GAD1, VGluT2, and CRF mRNA expression. Tiled images were converted to JPG2 files using Microfile+ software, kindly provided by Microbrightfield Biosciences. JPG2 images for each section were imported into Neurolucida software (Microbrightfield Biosciences). One investigator (J.L.F.) conducted mapping studies. Using the tracing tool, the boundaries of the CeLcn, CeM, SLEAc, BSTLP, and BSTLcn and VP were drawn over each section using adjacent immunostained sections for various markers, aligning landmarks, such as blood vessels and fibers tracts.

Once drawn, traced contours were hidden, and each channel was optimized independently under higher magnification using the “Zoom” tool. “Branched tree” RNA label is seen as small puncta that are frequently so dense that, in high-expressing cells, they form a confluence that completely fills the soma and/or nucleus (DAPI-positive confirmation). A positive labeled cell had labeled transcript in either the soma or nucleus, or both. Labeled neurons in each channel were manually marked in each section, with the other channels turned off. GAD1- and VGAT-mRNA were coexpressed in all cells examined; therefore, VGAT was chosen for quantification. Markers for CRF-mRNA-labeled cells (magenta), VGAT-labeled cells (green), and VGluT2-labeled cells (light orange) were sized at 30 µm, and then manually placed on labeled cells in each channel.

After separate markers were placed for each transcript in individual channels, contours were turned on, duplicates of each section were created, and individual maps of each subregion on the section were created for quantification and assessment of colocalization using a semiautomated method. We chose a marker overlap distance of 50 µm for automated colocalization, after preliminary trials. On subregion maps, total markers for each mRNA were checked under high power using the Zoom tool and recorded. Then, the “colocalization” tool was applied in sequence, first to triple-labeled cells (CRF, VGAT, VGluT2, marked with a “+”) and then to remaining double-labeled cells (CRF-VGAT and CRF-VGluT2 marked with “○” and “□,” respectively). Automated “colocalization” of markers was then checked by surveying random sites across all subregions. The total number of double- and triple-labeled cells was calculated for each subregion across four sections and summed. In a second set of analyses, the number of VGAT- and VGluT2-mRNA-labeled neurons in each subregion was assessed, without respect to CRF mRNA content. Colabeled VGAT- and VGluT2-mRNA cells were quantified. Cellular data are expressed as the proportion of labeled neurons within the entire region and in specific subregions.

## Results

### The CEA and its subdivisions

**Figure 1.**
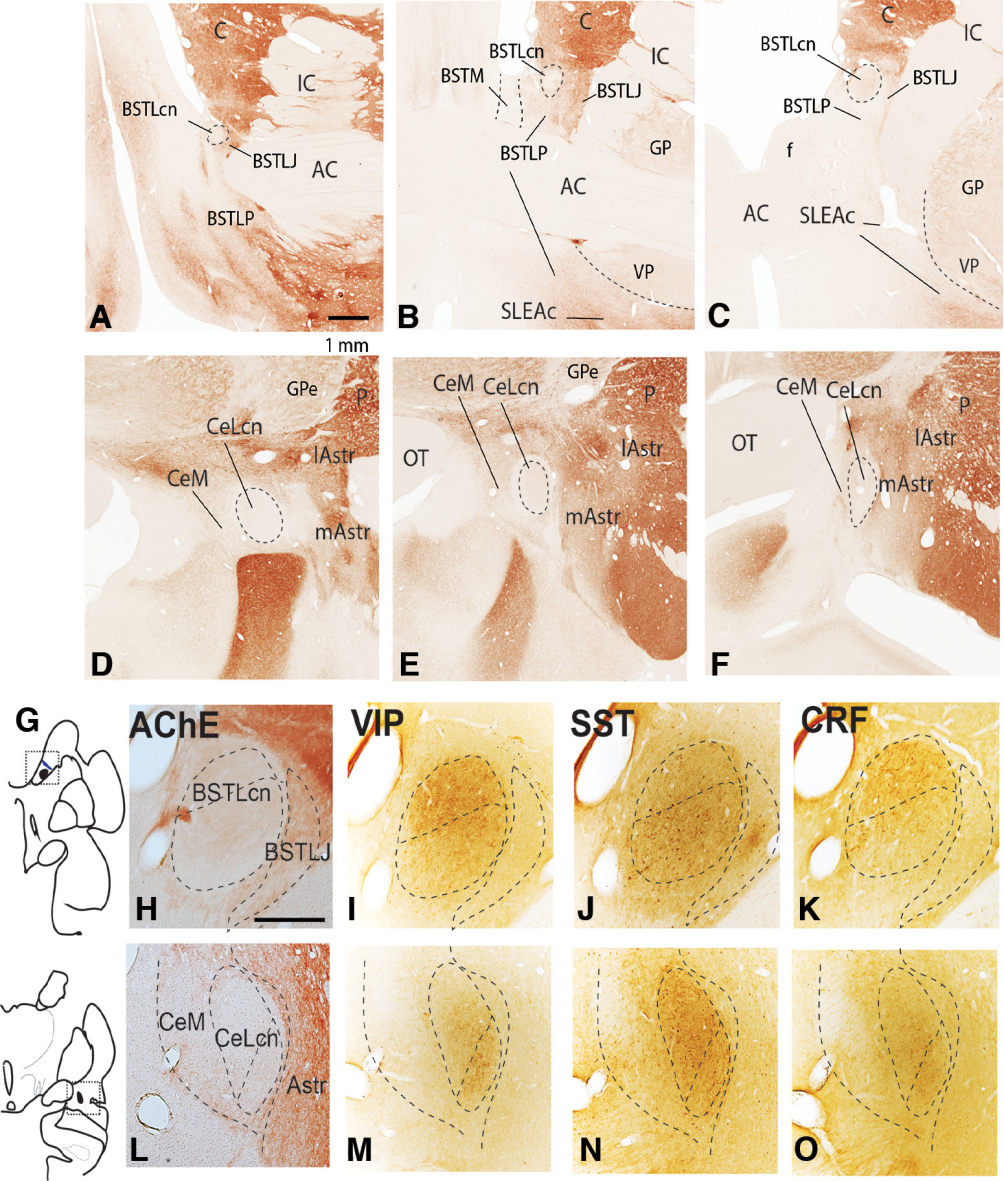
AChE activity in BSTL (***A–C***) and CeN (***D–F***) subdivisions. The BSTLcn and CeLcn have lowest levels of AChE staining; the BSTLP, SLEAc, and CeM have light activity; and the striatal-like BSTLJ and Astr (both medial and lateral) have moderate activity. These latter subdivisions transition with the striatum laterally. Scale bar, 1 mm. AChE and neuropeptide activity in the BSTLcn (***H–K***) and CeLcn (***L–O***), seen in adjacent sections. Scale bar, 1 mm. Panel ***G*** shows location of the BSTLcn (top) and and CeLcn (bottom). AC, Anterior commissure; Astr, amygdalostriatal area; BSTLcn, bed nucleus of the stria terminalis, lateral central subdivision; BSTLJ, bed nucleus of the stria terminalis, lateral juxtacapsular subdivision; BSTLP, bed nucleus of the stria terminalis, ventral posterior subdivision; BSTM, bed nucleus of the stria terminalis, medial subdivision; C, caudate nucleus; CeLcn, central nucleus, lateral central subdivision; CeM, central nucleus, medial subdivision; f, fornix; GP, globus pallidus, GPe, globus pallidus, pars externa, IC, internal capsule; lAstr, lateral amygdalostriatal area; mAstr, medial amygdalostriatal area; P, putamen; SLEAc, sublenticular extended amygdala, central subdivision.

The CEA spans a rostrocaudal distance of ∼6-7 mm in the monkey, beginning in the forebrain region just caudoventral to the shell of the nucleus accumbens, and extending through the ventral basal forebrain to the caudal central nucleus. Across species, the CEA has been considered a “variant” of the striato-pallidal system, with some atypical features that make it unique, including a high level of neuropeptides and long intrinsic interconnections from central nucleus to bed nucleus of the stria terminalis (Alheid and Heimer, 1988; Oler et al., 2016). In addition, “symmetrical subdivisions” based on histochemical and cellular features, at the rostral and caudal poles, have been described in both rodent and human brain (De Olmos, 1990; Heimer et al., 1999; McDonald, 2003) (Fig. 1*A–F*). The VP consists of the ventromedial globus pallidus, which stretches caudally to medial regions of the GPi.

### BSTLcn and CeLcn

**Figure 2.**
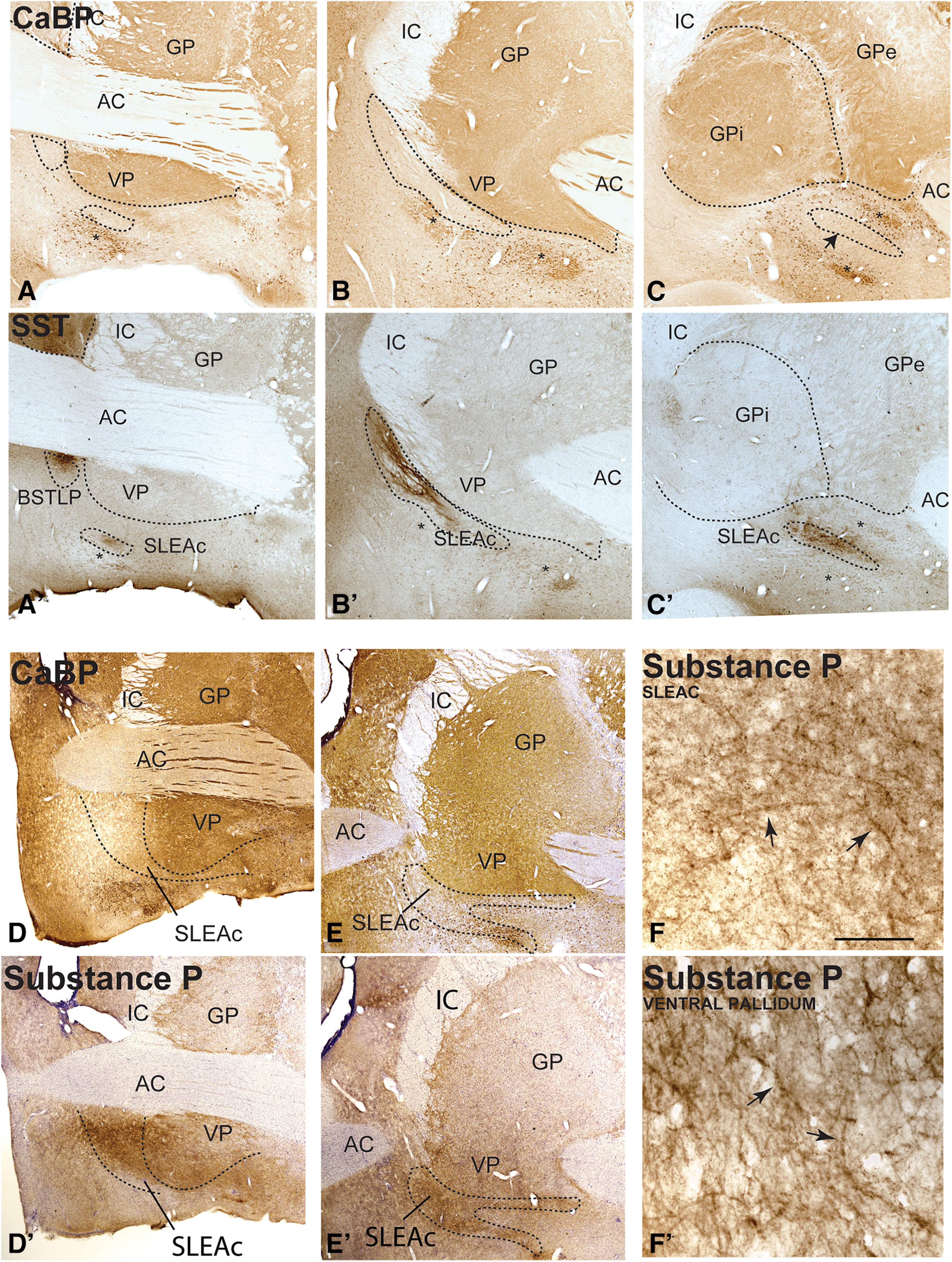
***A–C***, CaBP-IR in rostrocaudal sections through the ventral forebrain is classically high in the pallidal structures, and lower in the SLEAc and other CEA structures. The cholinergic neurons are strongly CaBP-IR (asterisks). ***A′–C′***, Sections adjacent to those in ***A–C***, immunostained for SST, which avoids the pallidum. ***D***, ***E***, Sections through the rostral and caudal VP (GPi), immunostained for CaBP to show pallidal boundary. ***D′***, ***E′***, Adjacent sections with SP IR showing that SP-labeled fibers traverse the SLEAc and ventral pallidal boundary at both rostral and caudal levels. ***F***, ***F′***, Higher-power photomicrographs represent “wooly fibers” in both the SLEA (***F***) and VP (***F′***). Arrows point to several examples. Scale bar, 100 μm.

The BSTLcn and CeLcn subdivisions “mirror” each other with low levels of AChE, and relatively high densities of peptide-IR (Decampo and Fudge, 2013). VIP-, CRF-, and SST-IR are higher in both the BSTLcn and CeLcn, compared with surrounding subregions (Fig. 1*H–O*). CeM and BSTLP:. The CeM and BSTLP have slightly higher levels of AChE than the CeLcn and BSTLcn. SST-IR serves as a distinguishing feature with the globus pallidus (Fig. 1). SLEAc (Fig. 2): The SLEAc sits between the CeM caudally, and the BSTLP rostrally, and there is no clear histochemical or cellular boundary between them. The SLEAc is therefore defined by its location under the globus pallidus, and it is not clearly a separate region in the CeM-SLEAc-BSTLP continuum. SST-IR distinguishes SLEAc from the VP (Decampo and Fudge, 2013). Cholinergic cell islands, which are not part of the CEA, are lodged in the SLEAc. They are CaBP-positive, and avoided by SST-labeled fibers (Chang and Kuo, 1991) (Fig. 2*A–C*,*A′-C′*, asterisks). VP: The calcium binding protein, CaBP, demarcates the globus pallidus, including the VP (Cote et al., 1991). Although SP-immunoreactive fibers, including “wooly fibers” (Haber and Watson, 1985), are classically considered a “marker” of the VP in rats (Zahm et al., 1996), in the monkey, SP-immunoreactive fibers broadly overlap both the VP (CaBP-positive) and also the SLEAc (CaPB-negative, SST-positive; Fig. 2*D*,*E*,*D′*,*E′*) (Haber et al., 1990a).

### Distribution of CRF-IR neurons in the CEA and VP

**Figure 3.**
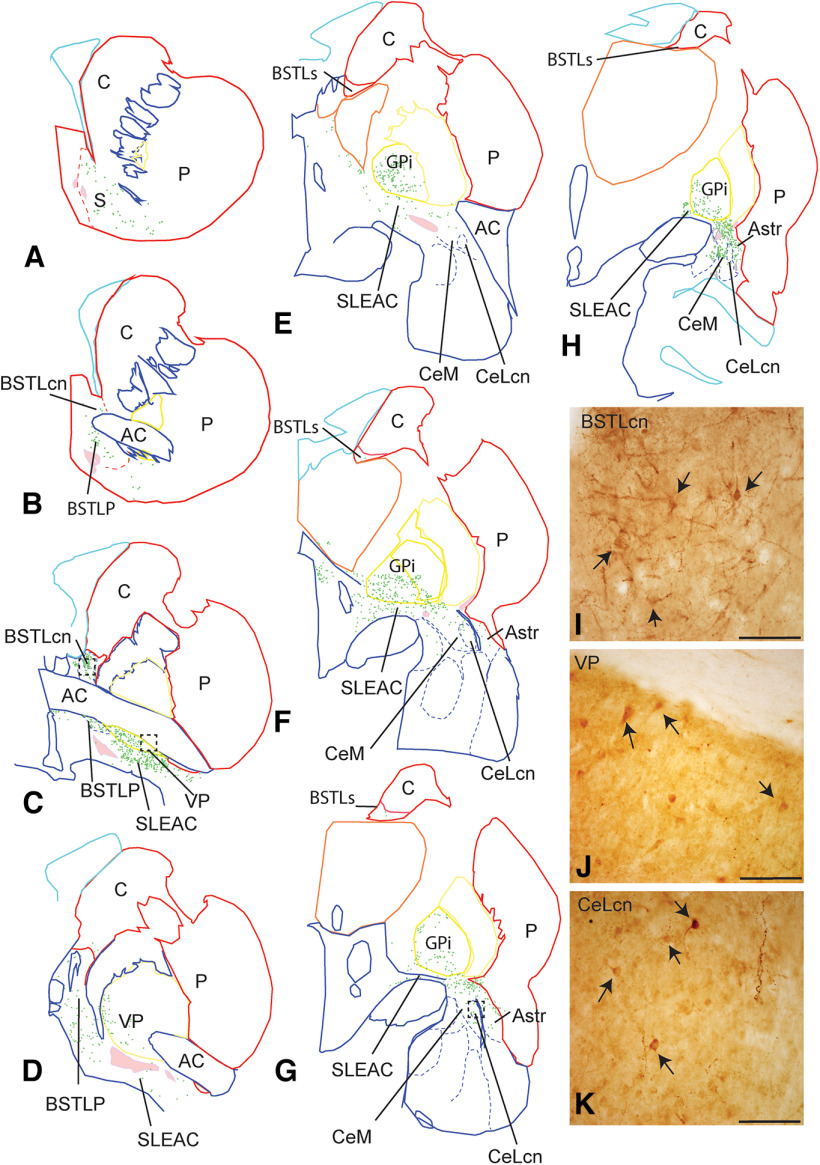
***A–H***, Distribution of CRF-immunoreactive neurons in the nonhuman primate CEA and VP subregions. Labeled neurons are distributed more broadly than previously reported, and extend from the ventral striatum through the caudal central nucleus. For clarity and to reduce abbreviations, colored lines indicate various surrounding structures: red represents striatum; cyan represents ventricles; yellow represents globus pallidus; orange represents thalamus; pink represents cholinergic cell clusters; green represents CRF-positive cells. ***I***, High-power micrograph of CRF-labeled cells and processes in the BSTLcn, corresponding to boxed area in ***C***. ***J***, High-power micrograph of CRF-labeled cells and processes in the VP, in the boxed area in ***C***. ***K***, CRF-labeled cells and fibers in the CeLcn, in region of boxed area in ***G***. Scale bars, 100 µm. AC, Anterior commissure; Astr, amygdalostriatal area; BSTLcn, bed nucleus of the stria terminalis, lateral central subdivision; BSTLJ, bed nucleus of the stria terminalis, lateral juxtacapsular subdivision; BSTLP, bed nucleus of the stria terminalis, ventral posterior subdivision; C, caudate nucleus; CeLcn, central nucleus, lateral central subdivision; CeM, central nucleus, medial subdivision; P, putamen; SLEAc, sublenticular extended amygdala, central subdivision.

CRF-positive cell bodes are found along the entire rostrocaudal extent of the CEA (Fig. 3*A–H*). The majority of CRF-labeled cells were small and densely stained. However, a somewhat surprising finding was the relatively large distribution of moderately stained large neurons in the VP, which continued into the caudal VP, pars interna (GPi). In CEA structures, CRF-labeled cells formed a continuum beginning in the caudal ventromedial shell of the ventral striatum, to enter the BSTLP (Fig. 3*A*,*B*). A cluster of labeled neurons were found in the BSTLcn (Fig. 3*I*), which entered the surrounding BSTLP and extended caudally to into the SLEAc (Fig. 3*C*,*D*). CRF-labeled neurons with high amounts of reaction product were densely concentrated in the BSTLcn, and embedded in dense neuropil (Fig. 3*I*). Labeled neurons were also found in the striatal-like BSTLJ. CRF-positive neurons in the SLEAc were mainly small and round (20-30 µm), and continued into the CeN (Fig. 3*F*,*G*), entering first in the anterior amygdaloid area and rostral CeM (Fig. 3*E*,*F*). In caudal sections, similarly small CRF-positive neurons occupied both the CeM and CeLcn (Fig. 3*G*,*K*) and were less densely concentrated than in the BSTLcn (Fig. 3*K*). There were also many CRF-positive neurons in the amygdalostriatal area and in GPi (Fig. 3*H*). There were CRF-positive neurons in the VP and also in the CaBP-positive internal segment of the globus pallidus. In the pallidum, CRF-positive neurons often had round or triangular-shaped morphologies (Fig. 3*J*).

### Distribution of VGAT- and VGluT2-mRNA in the CEA and VP

**Figure 4.**
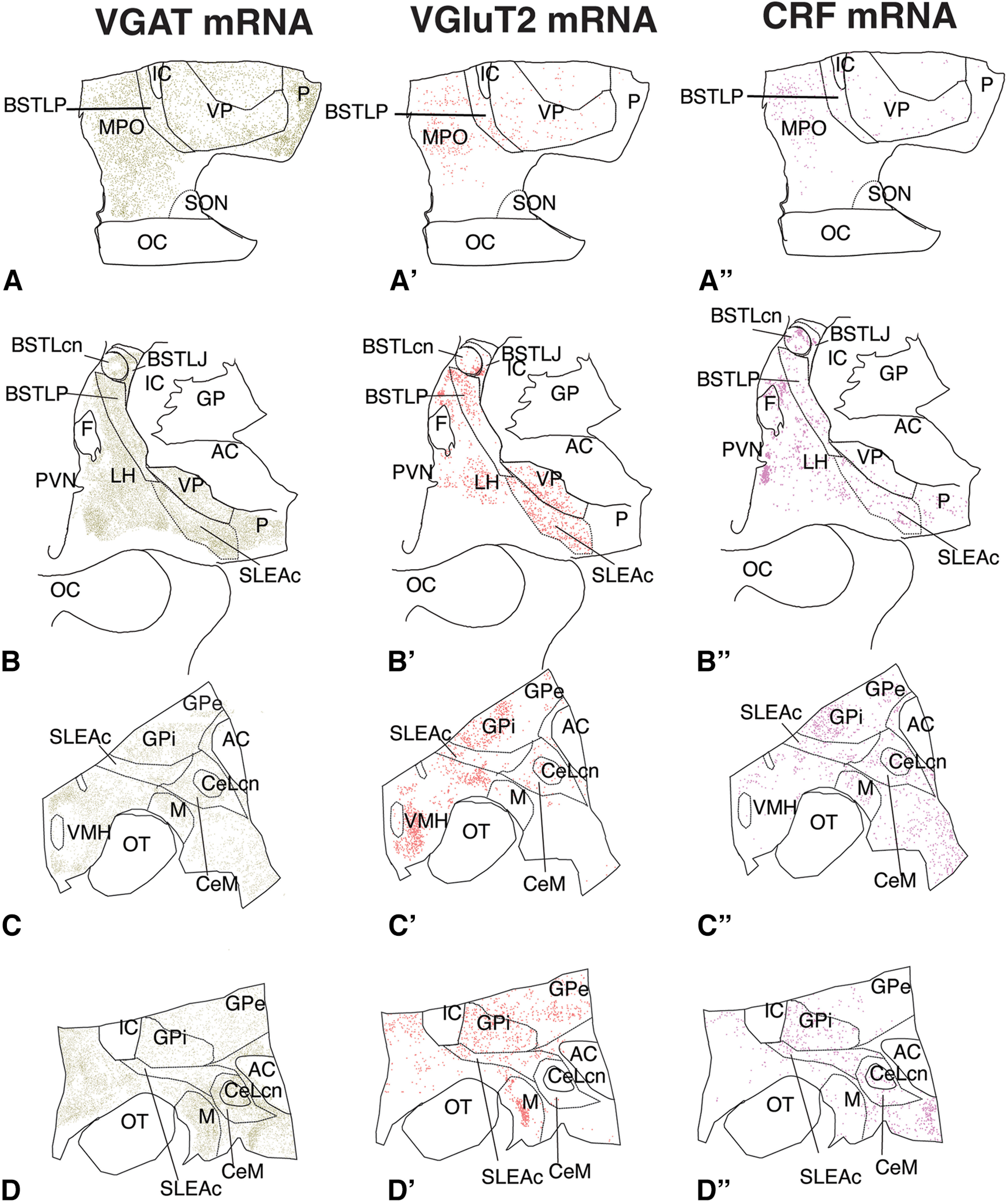
General distribution of all cells marked for VGAT mRNA (***A–D***, green), VGluT2 mRNA (***A′–D′***, orange), and CRF mRNA (***A″–D″***, magenta), in the CEA and VP, organized in rostrocaudal order. Some surrounding regions are also mapped. Colocalization of markers is not shown. AC, Anterior commissure; Astr, amygdalostriatal area; BSTLcn, bed nucleus of the stria terminalis, lateral central subdivision; BSTLJ, bed nucleus of the stria terminalis, lateral juxtacapsular subdivision; BSTLP, bed nucleus of the stria terminalis, ventral posterior subdivision; C, caudate nucleus; CeLcn, central nucleus, lateral central subdivision; CeM, central nucleus, medial subdivision; GPe, globus pallidus, external segment; IC, internal capsule; LH, lateral hypothalamus; M, medial nucleus of amygdala; MPO, medial preoptic area; OC, optic chiasm; OT, optic tract; P, putamen; PVN, paraventricular nucleus of the hypothalamus, SLEAc, sublenticular extended amygdala, central subdivision; SON, supraoptic nucleus; VMH, ventromedial hypothalamus.

General patterns of VGAT- and VGluT2-mRNA-containing neurons were first mapped through the CEA and VP (Fig. 4*A–D* and Fig. 4*A′-D′*, respectively). In the CEA, VGAT mRNA and VGluT2 mRNA-positive cells were often distributed in a “salt-and-pepper” manner, interspersed with one another. The vast majority of labeled neurons assessed through the CEA and VP as a whole were VGAT mRNA-positive (12,236, or 90%) (Table 2). Of all VGAT-positive neurons assessed throughout the CEA and VP, ∼11% (1405) of the total coexpressed VGluT2 mRNA. Of the VGluT2 mRNA cells detected overall, 52% (1405 of 2683) coexpressed VGAT mRNA. These results suggest that coregulation of VGluT2-positive neurons by GABA is a potential mechanism in a substantial proportion of excitatory neurons in the CEA and VP. We then examined the proportions of neuron types by subregion. While VGAT-single-labeled cells (presumptive inhibitory neurons) predominated in all CEA subregions and in the VP, the CeM and CeLcn had the highest proportions of single-labeled VGAT-mRNA-labeled cells (96%, and 97%, respectively). In contrast, the ventral pallidal subregions had the highest proportion of VGluT2-VGAT double-positive cells (776, or 24% of all labeled neurons); 69% of all VGluT2-positive in the VP neurons cocontained VGAT mRNA (776 of 1124).

**Table 2.** VGAT- and VGluT2-mRNA-labeled neurons^*a*^

	Total cells counted	VGAT total markers	VGluT2 total markers	VGAT/VGluT 2 double-labeled cells (% total)	VGAT single labeled cells (% total)	VGluT2 single-labeled cells (% total)
All regions	13,514	12,236	2683	1405 (11%)	10,831 (80%)	1278 (9%)
BSTLcn	738	691	136	89 (12%)	602 (82%)	47 (6%)
BSTLP	2704	2490	415	201 (7%)	2289 (85%)	214 (8%)
SLEAc	2899	2343	848	292 (10%)	2101 (71%)	556 (19%)
VP	3283	2935	1124	776 (24%)	2159 (66%)	348 (10%)
CeM	2657	2563	117	23 (1%)	2540 (96%)	94 (3%)
CeLcn	1260	1241	43	24 (2%)	1217 (97%)	19 (1%)

*^a^*Proportions of neurons expressing each marker, or both. Because of rounding to nearest integer, some proportions do not sum to 100%.

**Figure 5.**
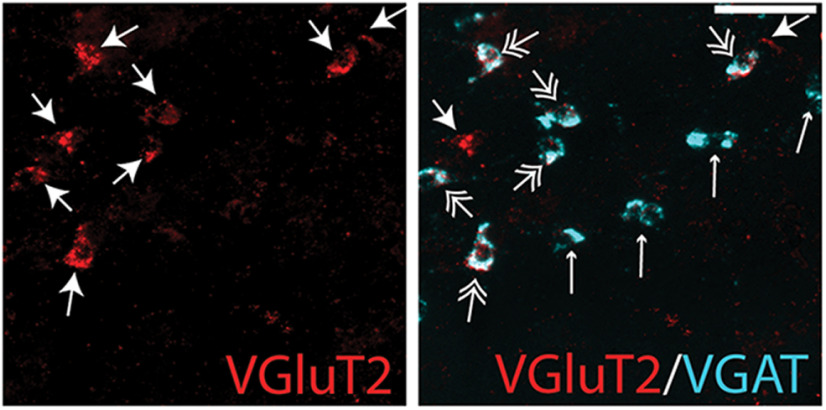
Left, VGluT2-mRNA-labeled cells in the VP (arrows, red). Right, VGAT-mRNA (cyan) frequently colocalizes with VGluT2-positive transcripts (double arrows, white) but sometimes does not (small white arrows). Scale bar, 100 μm.

Morphologically, VGluT2-positive neurons, both with and without VGAT mRNA colocalization, were generally larger than VGluT2-negative cells. In the SLEAc and BSTLP, double-labeled cells formed diagonally oriented chains that interdigitated among streams of small (20-30 µm), VGAT-mRNA single-labeled cells. In the VP, VGAT mRNA single-labeled and VGAT/VGluT2 double-labeled neurons were more spread apart, were larger, and resembled large pallidal cells (30-50 µm) (Fig. 5).

**Figure 6.**
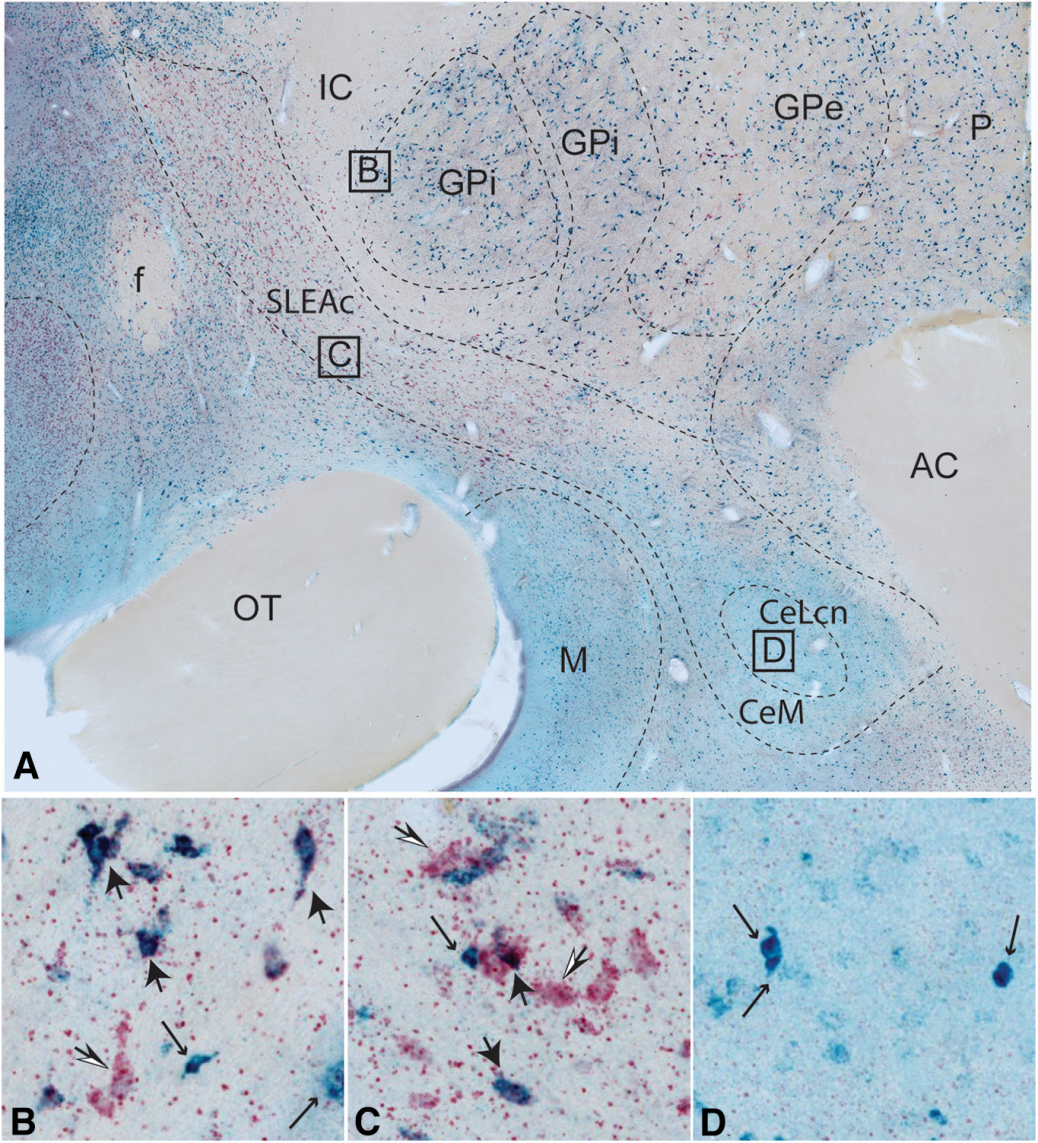
***A***, Macroscopic view of 2-plex colorimetric ISH for GAD1 (blue) and VGluT2 (red), at the level of the central nucleus and caudal SLEAc. Purple represents double-labeled neurons. Bright blue represents GAD1-positive cells. Bright red represents VGluT2-positive cells. ***B***, Higher-power view of labeled neurons in the VP (GPi, boxed area in ***A***). VGluT2 (red, open arrows) and VGluT2/GAD (purple, large black arrows) and GAD (cyan, small arrows)-positive neurons are intermingled. ***C***, Higher-power view of labeled neurons in the SLEAc (boxed area in ***A***). Intermingled VGluT2 (red), VGluT2/GAD (purple), and GAD-positive (cyan) neurons. ***D***, High-power view of neurons in the CeLcn (boxed area in ***A***). GAD-positive neurons (cyan) dominate, and are generally smaller than in the SLEAc and VP.

The relative distribution and morphology of neurons in 4-plex labeled sections were cross-referenced to sections visualized using 2-color GAD1/VGluT2 mRNA labeling (Fig. 6). As in the FISH-labeled sections, admixtures of GAD1 (cyan), VGluT2 (light red), and GAD1/VGluT2 mRNA (purple) labeled cells were present in the BSTLP (not shown), SLEAc, and VP (Fig. 6*B*,*C*). VGluT2-labeled cells (red, black/white arrows) and GAD1/VGluT2-labeled cells (purple, large black arrows) tended to be larger (30-50 µm) than GAD1-mRNA single-labeled cells (cyan, small black arrows), consistent with observations using fluorescent methods. In contrast, the predominant cell type in the BSTLcn (not shown) and central nucleus (Fig. 6*D*) was GAD1-positive (cyan, putative GABAergic, small arrows) neurons supporting data from FISH experiments.

### CRF mRNA colocalization with VGluT2 and VGAT mRNA

**Figure 7.**
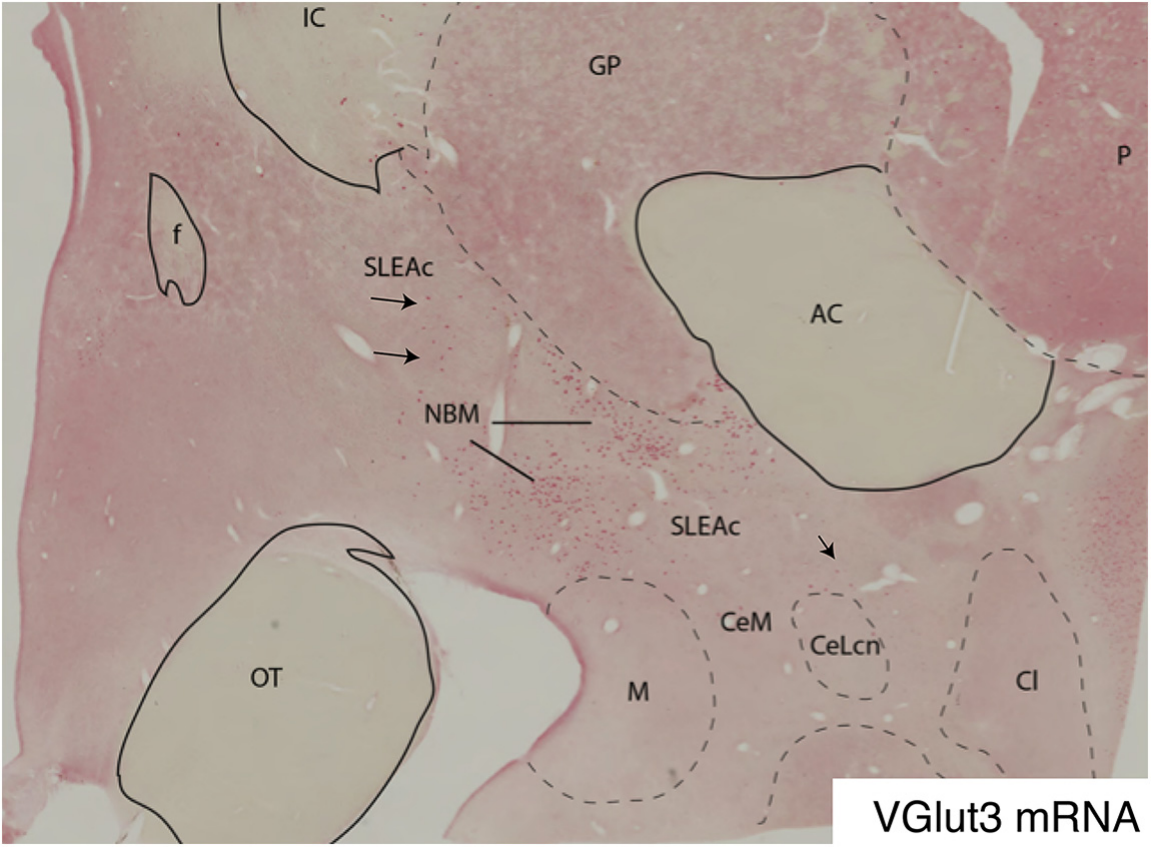
Single-labeling of VGluT3 mRNA-labeled cells in the basal forebrain. The VGluT3 mRNA-positive cells in the nucleus basalis of Meynert (NBM), and only scattered labeled cells in the SLEAc (arrows).

The distribution of single-labeled CRF-mRNA-positive cells was similar to that found for CRF-IR cells (Figs. 3, 4*A″-D″*). While ISH and FISH are not typically useful for describing cell morphology, the increased specificity and sensitivity of “branched” tree technology permit somewhat better cellular resolution (F. Wang et al., 2012). In the CEA, CRF-mRNA-positive neurons had heterogeneous sizes and morphologic features, while those in the VP tended to be larger, mirroring the histochemical studies; ∼11% of total VGluT2 and/or VGAT mRNA-labeled cells counted in the CEA and VP cocontained CRF mRNA (1525 of 13,514 neurons). Slightly <1% of CRF mRNA-labeled cells had neither VGAT nor VGluT2 mRNA colocalization (127 CRF-labeled neurons of 13,641 cells marked) (Fig. 7).

In the CRF-labeled population taken as a whole, the majority (87%) coexpressed VGAT mRNA (Table 3, all regions). However, many of the CRF/VGAT mRNA-labeled cells also coexpressed VGluT2 mRNAs (40%). Within the entire CEA and VP, 47% of CRF-positive cells expressed VGAT mRNA only, 40% expressed VGAT/VGluT2 mRNA, a small minority (5%) coexpressed only VGluT2, and a small proportion (8%) were not colabeled with either VGluT2 or VGAT mRNA.

**Table 3.** CRF mRNA expression in VGAT-, VGluT2-, and VGAT/VGluT2-positive cells^*a*^

	CRF total cells counted	CRF/VGAT double	CRF/VGluT2 double	CRF/VGAT/ VGluT2 triple	CRF single
All regions	1525	47% (714)	5% (77)	40% (607)	8% (127)
BSTLcn	123	74% (91)	1% (1)	10% (12)	15% (19)
BSTLP	367	54% (199)	4% (15)	33% (121)	9% (32)
SLEAc	285	54% (154)	7% (19)	27% (78)	12% (34)
VP	553	17% (97)	7% (40)	67% (371)	8% (45)
CeM	149	85% (126)	1% (1)	7% (11)	7% (11)
CeLcn	64	73% (47)	2% (1)	16% (10)	9% (6)

*^a^*Proportions of neurons expressing each marker, or both. Because of rounding to nearest integer, some proportions do not sum to 100%.

**Figure 8.**
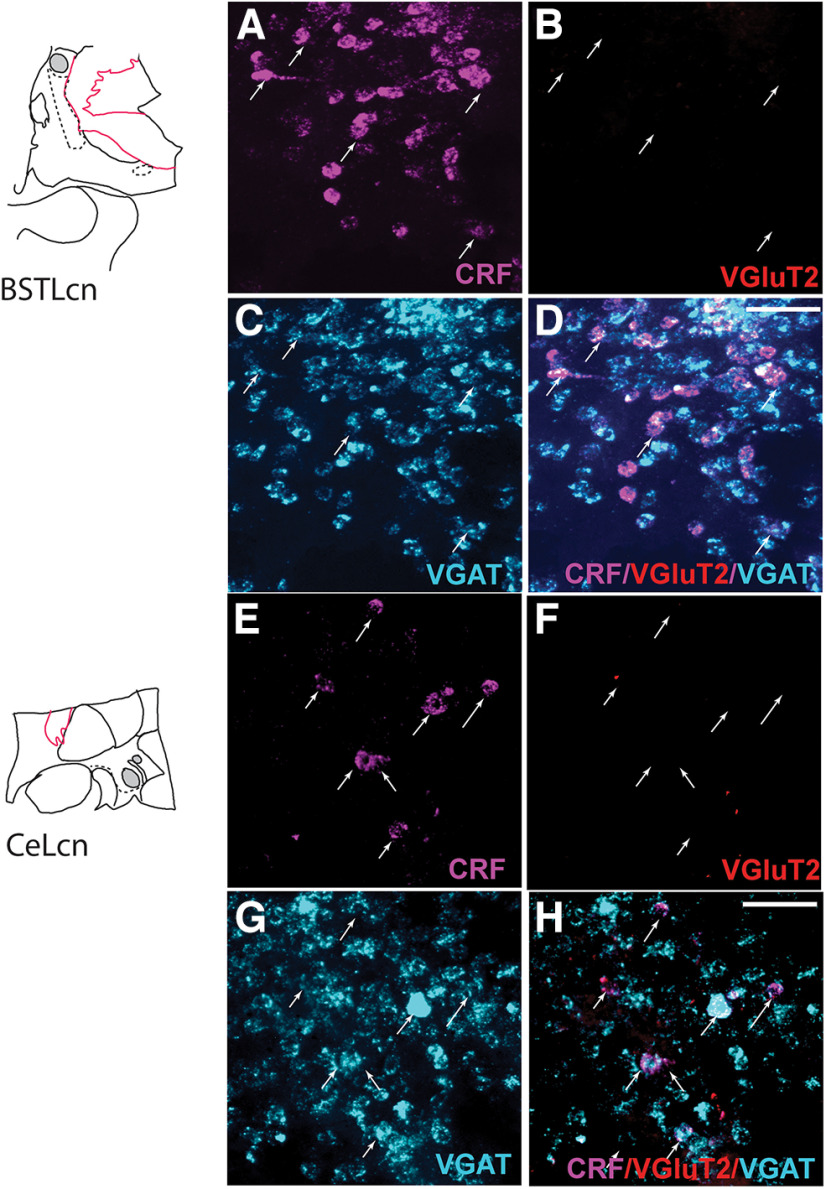
***A–D***, Neurons in the BSTLcn seen in channels for CRF, magenta (***A***), VGluT2, red (***B***), VGAT, cyan (***C***), and CRF/VGluT2/VGAT (***D***). Arrows track CRF mRNA-containing neurons through each channel. Scale bar, 100 μm. ***E***, ***F***, Neurons in the CeLcn seen in channels for CRF, magenta (***E***), VGluT2, red (***F***), VGAT, cyan (***G***), and CRF/VGluT2/VGAT (***H***). Arrows track CRF labeled cells in all panels. Scale bar, 100 μm.

The relative proportion of CRF-expressing cell types varied by subregion. The majority of CRF-mRNA neurons in the BSTLcn, CeM, and CeLcn contained only VGAT-mRNA (74%, 85%, 73%, respectively) (Table 3; Fig. 8*A–D*, BSTLcn; Fig. 8*E*,*F*, CeLCn). CRF/VGAT-labeled cells in these regions were morphologically small and round, in the 20-30 µm range (Fig. 8*A*,*C*,*D* and Fig. 8*E*,*G*,*F*, arrows). Dense clusters of CRF/VGAT-labeled neurons were seen in the BSTLcn (Fig. 8*A–D*) (similar to the distribution of CRF-IR neurons, Fig. 3*C*,*I*), whereas in the CeLcn, CRF/VGAT-labeled cells were more dispersed (Fig. 8*E–H*), also consistent with CRF protein labeling (Fig. 3*G*,*K*). There were relatively small complements of CRF/VGAT neurons also cocontaining VGluT2 mRNA (10%, 7%, 16%), which we term “multiplexed” (CRF/VGAT/VGluT2).

**Figure 9.**
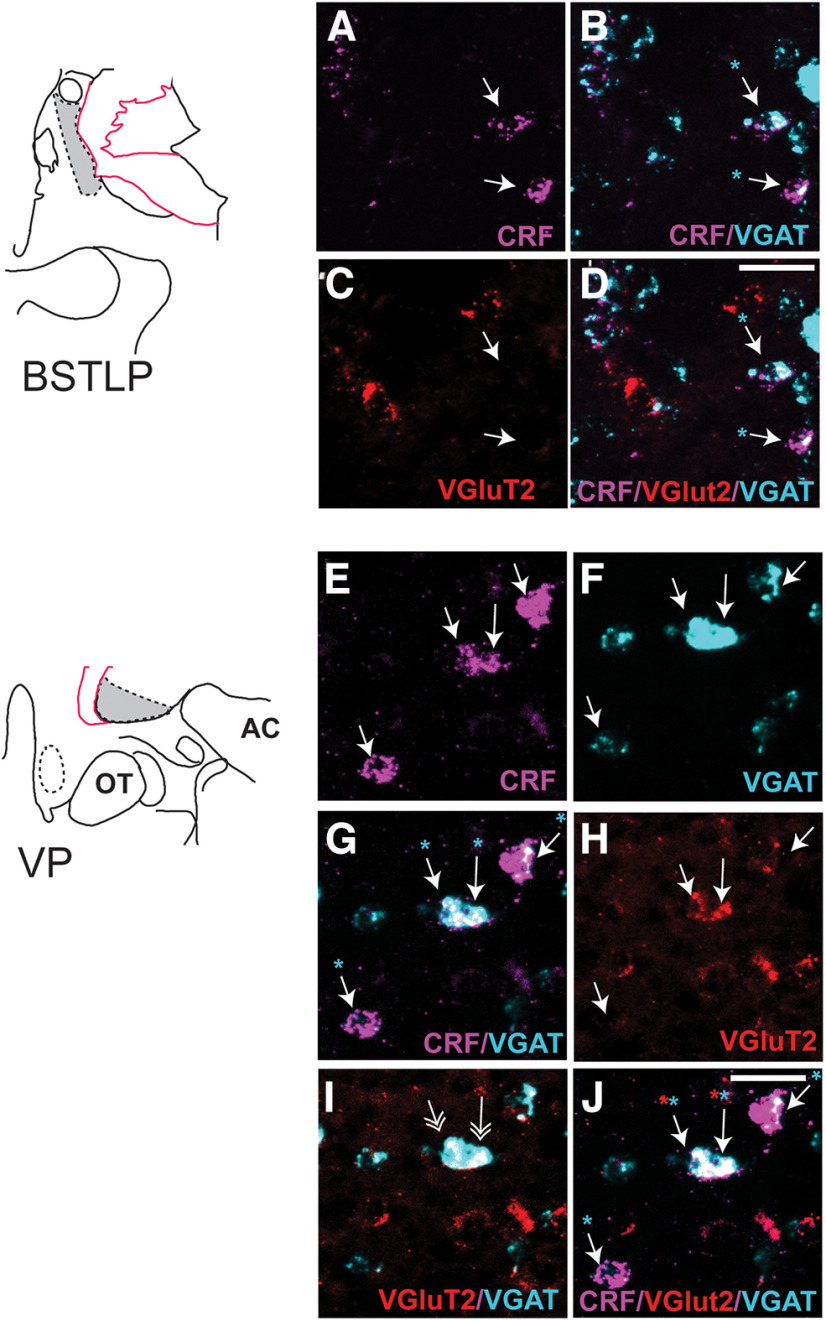
***A–D***, Higher magnification of neurons in the BSTLP labeled for (***A***) CRF, magenta, (***B***) VGAT, cyan and CRF, magenta, (***C***) VGluT2, red, and (***D***) CRF/VGluT2/VGAT mRNAs (magenta, red, cyan). Arrows track CRF mRNA-containing neurons (***A***) through each channel. Scale bar, 50 μm. ***E–J***, High-magnification views of VP neurons visualized in channels for (***E***) CRF, magenta, (***F***) VGAT, cyan, (***G***) CRF, magenta and VGAT, cyan, (***H***) VGluT2, red, (***I***) VGluT2, red and VGAT, cyan with double arrowheads showing double-labeled cells, and (***J***) CRF, magenta/VGluT2, red/VGAT, cyan. Single arrows track CRF mRNA-containing neurons in ***E***. ***J***, Cyan and red asterisks, respectively, denote VGAT and VGluT2 labeling in these cells. Scale bar, 50 μm.

**Figure 10.**
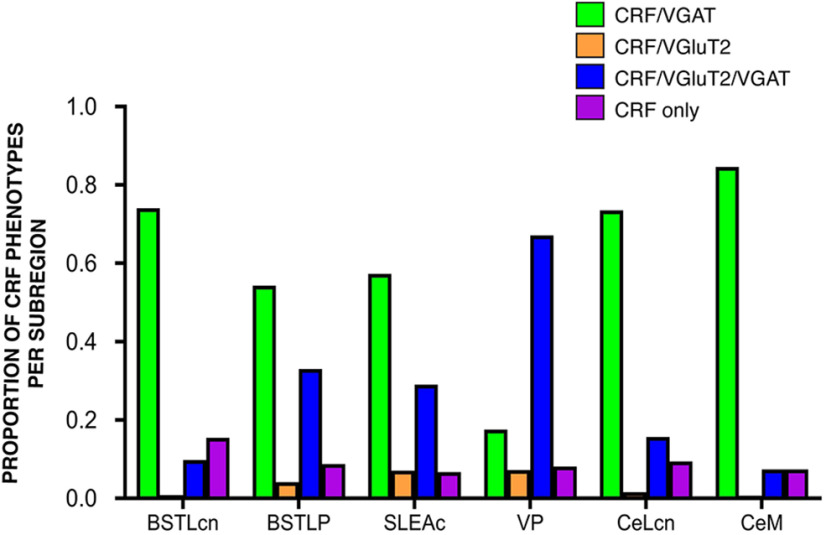
Histogram represents the differing proportions of CRF phenotypes in the CEA subregions and VP (details in Table 3). CRF/VGAT (light green), CRF/VGAT/VGluT2 (blue), CRF/VGluT2 (orange), and CRF alone (purple).

In contrast to the BSTLcn, CeM, and CeLcn, CRF-positive neurons in the BSTLP and SLEA were less likely to contain VGAT mRNA alone (54% of CRF-labeled cells in each subregion), with a higher proportion of CRF/VGAT/VGluT2-positive neurons in these subregions (33% and 27%, respectively) (Table 3; Fig. 9*A–D*, BSTLP). Neurons in the BSTLP and SLEAc were relatively heterogeneous, with small CRF/VGAT-labeled neurons typical of the BSTLcn and central nucleus, and also medium-sized, pallidal-like cells, which often were CRF/VGAT/VGluT2-positive. Last, the VP stood out for having larger triangular or round cell bodies, which were relatively widely spaced compared with those in the BSTLP and SLEAc (Fig. 9*E–H*, VP). The majority of CRF-positive neurons in the VP cocontained both VGAT and VGluT2 mRNA (67%) (Fig. 9*H*, arrows), with only 17% of CRF-positive neurons coexpressing VGAT-mRNA alone and 7% expressing VGluT2-mRNA alone (Fig. 9*H*, asterisks). The relative proportions of CRF phenotypes are presented in Figure 10.

## Discussion

There are several key findings in this study. First, CRF protein and mRNA-labeled cells had a similar distribution. Second, despite a dense cluster of CRF-labeled cells in the BSTLcn, the majority of CRF-IR neurons were broadly dispersed throughout the rest of the CEA, including in the BSTLP, SLEA, CeLcn, and CeM. This picture may be somewhat at odds with rodent models in which CRF-positive neurons are depicted as largely confined to dense clusters in BSTL or CeN. Surprisingly, the VP, both at classic rostral levels and its continuation into the ventromedial GPi, also contained CRF-expressing cell bodies, detected both with protein and mRNA assays. These neurons had a typical morphology of pallidal neurons with large triangular cell bodies (Parent, 1979).

Another finding is that the territory comprising the CEA and VP contains mixtures of cells expressing glutamate, GABA, or both (GABA/glutamate), with the relative proportions of each varying by subregion. While this is perhaps not surprising, the CEA and VP had long been considered primarily GABAergic structures. The advent of specific glutamatergic and GABAergic cell markers has revealed a more complex cellular organization in rodents, which we appreciated in the primate (Hur and Zaborszky, 2005; Poulin et al., 2008, 2009; Kudo et al., 2012; Root et al., 2018). We found that the majority of mapped cells in the CEA and VP as a whole were GABAergic, although relative proportions of GABA/glutamatergic neurons, and “pure” glutamatergic neurons, varied by subregion. Our results are broadly consistent with findings in the rat where, for example, the “anterior” bed nucleus (monkey BSTLcn) and central nucleus have dense GAD67 mRNA and little VGluT2 mRNA, while many cells in the more “posterior” bed nucleus (primate BSTLP) and SLEAc are VGluT2 mRNA-positive (Hur and Zaborszky, 2005; Poulin et al., 2008, 2009). Because these studies did not use double-labeling, the extent of VGAT/VGluT2-labeled cells was not reported. However, recent mouse work supports VGAT/VGluT2 subpopulations in the BSTL (Soden et al., 2022). As per our finding in monkey VP, the rodent “entopeduncular nucleus” (GPi homolog) (Zander et al., 2010; Shabel et al., 2014; Root et al., 2018; S. Kim et al., 2022), and monkey GPi (Conte-Perales et al., 2011) have a high proportion of VGluT2/VGAT-mRNA-positive neurons. These pallidal findings suggest that an intricate excitatory/inhibitory balance is maintained at the cellular level in these regions (Zander et al., 2010; Tritsch et al., 2016; Granger et al., 2017; Root et al., 2018).

**Figure 11.**
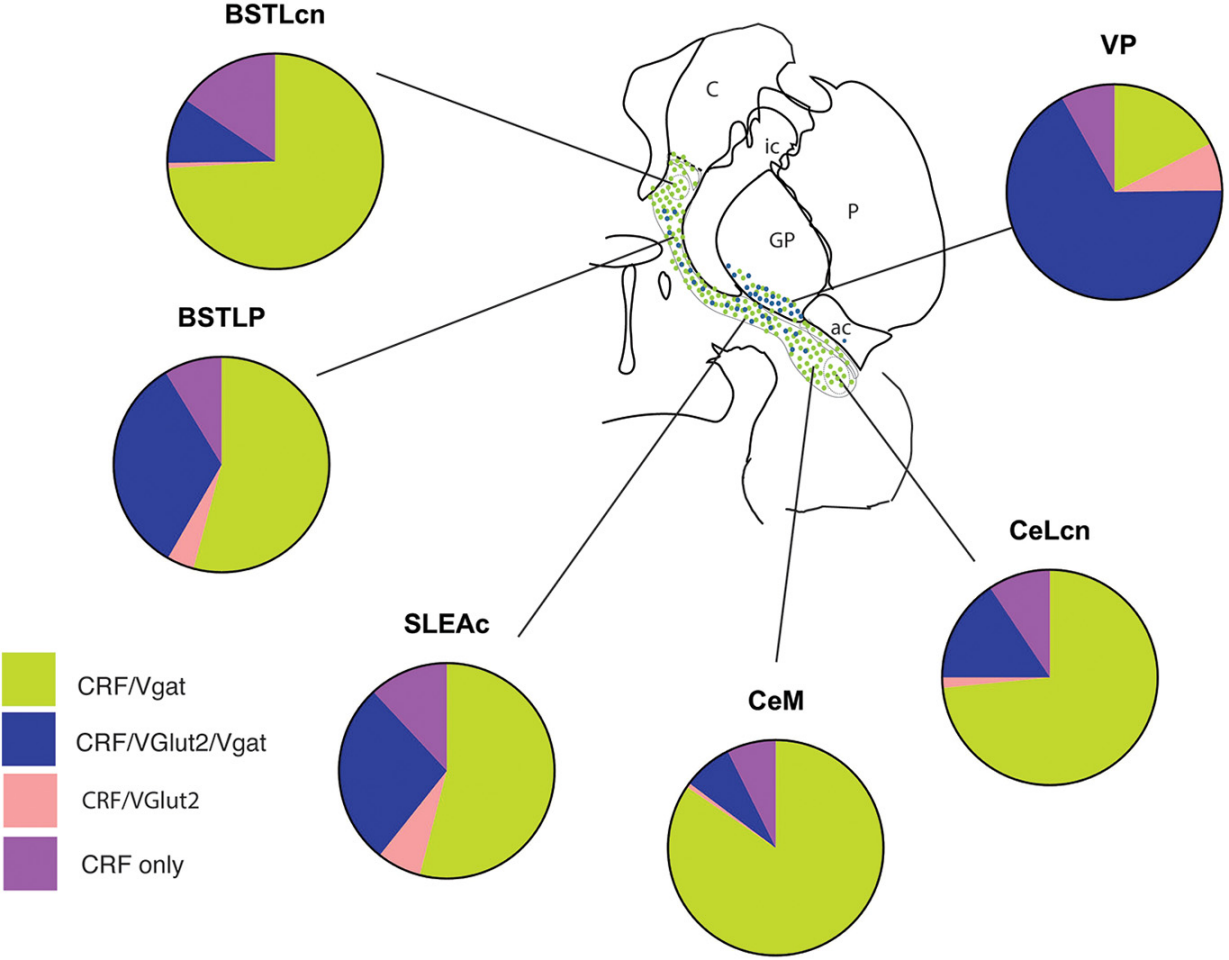
Schematic of CRF phenotype distribution across the CEA and VP by anatomic location. Subregions have differing admixtures of CRF phenotypes, including CRF/VGAT (light green), CRF/VGAT/VGluT2 (blue), CRF/VGluT2 (orange), and CRF alone (purple).

We also found that CRF expression follows primary transmitter patterns across CEA subdivisions and the VP. There are relatively homogeneous populations of CRF/GABA neurons at the “poles” of the CEA (i.e., the BSTLcn and CeLcn/CeM) (Fig. 11, green). In contrast, CRF-expressing neurons in the BSTLP and SLEAc are more heterogeneous with respect to primary transmitter phenotype, with an “excitatory” CRF subpopulation that colocalizes VGAT transcripts (Fig. 11, blue). This trend was even more pronounced in the VP subregions, where “multiplexed” CRF/VGAT/VGluT2 neurons were the rule rather than the exception. Gradients of cellular phenotypes across these various regions are broadly consistent with the development of CEA and VP, where a “mosaic” of progenitors migrate in from the lateral and medial ganglionic eminences, and preoptic area, to differentially settle into overlapping cellular corridors (Puelles et al., 2000; Nobrega-Pereira et al., 2010; Waclaw et al., 2010; Bupesh et al., 2011; Vicario et al., 2015).

### CRF neuronal heterogeneity in CEA and VP circuitry

Across species, the CEA develops from the “subpallial” structures cited above, eventually “stretching” along the rostrocaudal axis as the brain enlarges and incoming fiber tracts divide the ventral forebrain. Similar combinations of progenitors form the BSTLcn and CeLcn “poles” of CEA, and presumably give rise to similar patterns of CRF/GABA neurons (Veinante and Freund-Mercier, 1998; Kudo et al., 2012; Dabrowska et al., 2013, 2016; Partridge et al., 2016; Pomrenze et al., 2019; Soden et al., 2022). However, multiple other peptides are found in BSTLcn and CeLcn/CeM neurons (J. Kim et al., 2017; Soden et al., 2022). Our data in the monkey suggest that these peptides are mainly expressed against a GABAergic background in the BSTLcn and CeLcn/CeM. CRF neurons in the BSTLcn and CeLcn/CeM send not only extrinsic outputs but self-regulate by way of intrinsic collaterals (Marcinkiewcz et al., 2016; Sanford et al., 2017).

In contrast, the monkey BSTLP and SLEAc have mixtures of CRF neuronal phenotypes, consisting of GABAergic, glutamatergic, and “mixed” populations. In rodents, the BSTLP exhibits glutamatergic and GABAergic postsynaptic currents, consistent with the cellular heterogeneity in that species (Georges and Aston-Jones, 2001; Jennings et al., 2013; Daniel and Rainnie, 2016). Furthermore, driving BSTLP VGluT2 versus VGAT neurons in male mice using optogenetic techniques is anxiogenic and anxiolytic, respectively, and associated with differential midbrain targets (Jennings et al., 2013). CRF expression in these respective subcircuits remains to be examined but may be one way that gain is set in opposing circuits during natural behaviors. Of course, the BSTLP and SLEAc also target many other hypothalamic and brainstem nuclei, and understanding of CRF cell type-specific effects is emerging (Giardino and Pomrenze, 2021).

We found that CRF-containing VP neurons are frequently “multiplexed,” expressing both VGLuT2 and VGAT, and suggesting a mechanism of “corelease” as has been reported in rodent (Zander et al., 2010; Shabel et al., 2014). In addition to ventral striatal inputs, the entire monkey VP and GPi receive afferent inputs from the subthalamic nucleus, pedunculopontine nucleus, and midbrain dopamine cells (Lavoie and Parent, 1994; Y. Smith et al., 1994; Shink and Smith, 1995). In monkey, retrograde injections into the lateral habenula result in a broad “corridor” of retrogradely labeled cells encompassing both the ventromedial pallidum and adjacent “substantia innominata” (BSTLP and SLEAc) (Parent and De Bellefeuille, 1982). We found that CRF-multiplexed neurons overlap this entire region, where efferents to the lateral habenula play a role in reward/punishment signaling (Parent and De Bellefeuille, 1982; Parent et al., 2001; Hong and Hikosaka, 2008; Conte-Perales et al., 2011). We speculate that CRF may modulate the balance of excitatory/inhibitory output in these circuits, and that it can be influenced by stress (Shabel et al., 2019).

### CRF's role with primary transmitters

CRF is a widely expressed neuropeptide that functions as a comodulator of fast transmitters, and is expressed in both symmetric (putative inhibitory) and asymmetric (putative excitatory) terminals (Gray et al., 1982; Hendry et al., 1984; Tagliaferro and Morales, 2008). At the synapse, CRF influences postsynaptic membrane properties, and is also involved in synaptic plasticity (Aldenhoff et al., 1983; Liu et al., 2004). CRF's role in either LTP or LTD is circuit-specific, and depends on pathway-specific primary transmitters as well as postsynaptic CRF receptors (Miyata et al., 1999; H. L. Wang et al., 2000; Vandael et al., 2021). Consistent with this, CRF shapes synaptic strength by inducing structural changes at the presynaptic and postsynaptic membrane, in a timing- and duration-dependent manner (Chen et al., 2013; Gounko et al., 2013; Vandael et al., 2021). Therefore, the ability to examine CRF with its primary cotransmitter(s) in a circuit-specific manner is a fundamental step in untangling how CRF modulates downstream pathways, and its role in specific behaviors.

### Future directions

The regional diversity of CRF-mRNA cellular phenotypes found here is reminiscent of findings in the hypothalamus where CRF in GABAergic, glutamatergic, and multiplexed subpopulations maps onto specific regional distributions, suggesting modulation of specific microcircuits in male rodent models (Hrabovszky et al., 2005; Romanov et al., 2017). A future goal is to identify CRF-containing microcircuits issuing from the CEA and VP in the primate. Beyond identifying CRF phenotypes that participate in specific circuits, another question is whether CRF is subject to induction or amplification in differential cell types in the face of stress (Helmreich et al., 1999; McNally and Akil, 2002; Shepard et al., 2006), and whether this affects synaptic structure.

A limitation of our study is that we did not examine sex differences, which are an important issue in all work involving stress and CRF systems (for review, see Wellman et al., 2018). Developmental time points may also be critical for CRF expression and stress sensitivity across sexes (Yohn and Blendy, 2017; B. L. Smith et al., 2018). Importantly, structural and cellular sexual dimorphisms of medial (Allen and Gorski, 1990) and lateral extended amygdala nuclei exist in humans (Zhou et al., 1995), and may share similarities with rodents. Future work in young female macaques will therefore be important, as will studies involving perturbations of CRF in cell type-specific circuits in both sexes.
